# Investigation of Hepatic Blood Perfusion by Laser Speckle Imaging and Changes of Hepatic Vasoactive Substances in Mice after Electroacupuncture

**DOI:** 10.1155/2014/715316

**Published:** 2014-07-21

**Authors:** Xiao-jing Song, Dong Zhang, Shu-you Wang, Shun-yue Li

**Affiliations:** Department of Biomedical Engineering, Institute of Acupuncture and Moxibustion, China Academy of Chinese Medical Sciences, 16 Nanxiaojie, Dongzhimen, Beijing 100700, China

## Abstract

The study was conducted to observe the effect of electroacupuncture (EA) on hepatic blood perfusion (HBP) and vascular regulation. We investigated 60 male anesthetized mice under the following 3 conditions: without EA stimulation (control group); EA stimulation at Zusanli (ST36 group); EA stimulation at nonacupoint (NA group) during 30 min. The HBP was measured using the laser speckle perfusion imaging (LSPI). The level of nitric oxide (NO), endothelin-1 (ET-1), and noradrenaline (NE) in liver tissue was detected by biochemical methods. Results were as follows. At each time point, HBP increase in ST36 group was higher than that in the NA group in anesthetized mice. HBP gradually decreased during 30 min in control group. The level of NO in ST36 group was higher than that in NA group. The level of both ET-1 and NE was the highest in control group, followed by NA group and ST36 group. It is concluded that EA at ST36 could increase HBP possibly by increasing the blood flow velocity (BFV), changing vascular activity, increasing the level of NO, and inhibiting the level of ET-1 in liver tissue.

## 1. Introduction

Clinically, acupuncture has remarkable effects on various liver diseases. Acupuncture and moxibustion treatment can effectively improve immunity of patients with hepatitis and lower blood lipids of patients with fatty liver and improve their clinical symptoms. It also can relieve pain and reduce the side effects of radiotherapy and chemotherapy in patients with liver cancer, thus enhancing the quality of life and prolonging the life [1–3]. In addition, it is proved that acupuncture and moxibustion have favorable regulative actions on blood circulation in a multiway, multilevel, multilink, and multisubstance manner [4]. It is reported that acupuncture treatment can improve hemorheology and vasoactive substances, such as thromboxane A_2_(TXA_2_), prostaglandin I_2_(PGI_2_), endothelin (ET), and atrial natriuretic factor (ANF) [5]. Zhang et al. [6] reported that blood supply in the stomach of ischemia-reperfusion is improved and promoted by EA stimulation. Therefore, the increase of visceral blood perfusion (BP) is an important effect of acupuncture and a basis in the acupuncture treatment for visceral diseases [7, 8].

The hepatic circulation (HC) is very rich. Reports suggest that HBP have a significant association with visceral disorders. Leveson et al. [9] reported that gastrointestinal cancer patients with simultaneous liver metastasis exhibited a high hepatic arterial blood flow (BF). Leggett et al. [10] reported that colorectal cancer patients with simultaneous liver metastases revealed that the hepatic arterial BF was significantly increased and the portal BF was decreased. HBP can be a potential biomarker for predicting clinical progression or outcomes of cancer patients [11, 12]. In addition, computed tomography perfusion (CTP) had been successfully applied in a variety of clinical conditions of the liver. It was detected that HBP was decreasing in hepatocirrhosis condition [13, 14]. Therefore, we got the idea that HBP would be an important biomarker for understanding the mechanism of the effects of acupuncture on HBP for the use of acupuncture therapy in liver diseases. In this study, LSPI technique was used to display the HBP in mice before and after EA and analyze the time-effect relationships between EA and HBP. At the same time, the relationships between neurotransmitter, vasoactive substances, and the HBP changes were investigated in order to explore the mechanism of the EA effects on HC.

## 2. Materials and Methods

### 2.1. Animals and Groups

This study utilized 60 healthy adult male Kunming mice, weighing 21 ± 5 g, average age of 3 months, provided by the Animal Experiment Center, Academy of Military Medical Sciences (China). The 60 mice were randomly allocated to three groups: ST36 group, NA group, and control group, 20 mice in each group. All experimental procedures were approved by the Ethical Committee of Academy of Medical Sciences and were conducted in accordance with the internationally accepted principles for laboratory animal use and care.

### 2.2. Electroacupuncture

For the ST36 group, bilateral ST36 which was located at the posterolateral knee of hind limbs, about 2 mm below the fibular head, were stimulated with 32^#^ needle (0.18 × 13 mm) to 3 mm deep. Then, the needles were connected to the EA device (Hanshi Pain Healing Device, Hanshi-100A; Nanjing Jisheng Medical Technology Company, Nanjing, China). The stimulation time was 30 min, the current intensity was 5 V, and the pulse frequency was 50 Hz. For the NA group, the bilateral nonacupoints which were located at medial to ST36 and close to the margin of tibia were stimulated. And the method of EA was the same as that for ST36 group. For the control group, acupuncture was not done.

### 2.3. Preparation of In Vivo Mouse Hepatic Model

The mouse was anesthetized by intraperitoneal injection of 2% pentobarbital sodium (2.5 mg/kg, Sigma-Aldrich, St. Louis, USA) 30 min before preparation of in vivo mouse hepatic model. Under anesthesia, the mouse was fixed on a plank in supine position. Approximately 1 cm of linear incision below the xiphoid process and along the ventral median line was made. Then the liver tissue was exposed. The underneath liver lobe was separated and plainly placed on the top of a bracket above the abdomen.

### 2.4. Measurement of LSPI

The mouse model was placed in the experimental constant temperature box whose temperature was kept at 30–32°C and relative humidity was 80–90%. The liver lobe was placed about 28–30 cm below the laser scanner. The box was placed in a shielding chamber without direct sunlight, infrared radiation, and ventilation.

Moor-FLPI laser speckle perfusion (LSP) imager (Moor instruments Ltd, Axminster, UK) was used in this study. The scanning mode was the low density and 25 fps, the time interval was 1 s, exposure time was 20 ms, and 10 frames were continually scanned at each time point (10 frames were averagely processed into single frame to obtain the mean HBP at each time point). The instrument system can simultaneously record the LSP image and the digital coded image of the liver (actual positional image of the examined part). The two images were used to analyze the relationships between the BF distribution and the hepatic surface position.

For ST36 group and NA group, the HBP image was recorded before EA and every 5 min in 5 to 30 min during EA. For control group, the HBP image was recorded every 5 min in 0 to 30 min (Figure 5). Then the HBP images were saved and analyzed by the Image Review program of Moor-FLPI-V2.0 software. The location, range, and degree of the HBP of three groups were compared at each time point. The round region of interest (ROI) with the same area in each LSP image was selected for measuring the HBP.

### 2.5. Determination of Hepatic Vascular Regulators

After the HBP image was recorded, the scanned lobe of liver (about 0.5 g) was taken and homogenized with adequate 0.89% cold saline and centrifuged. Then, the supernatant was taken to detect the level of NO with nitric acid reductase and the level of ET-1 with radioimmunoassay (The NO & ET-1 Assay Kits, Beijing Sinouk Institute of Biological Technology, China; r-911 Radio-immune Counter, Corporation of Industry and Commerce, University of Science and Technology of China, China) and the level of NE with enzyme-linked immunoabsorbent assay (NE Assay Kit, R&D Systems, USA; STET FEX 2100 Enzyme Labeling Meter, Awareness Technology Inc, USA).

### 2.6. Statistical Analysis

The mean HBP was, respectively, calculated with perfusion unit (PU for short) as the unit. The value of HBP at each time point was expressed as mean ± standard deviation. SPSS13.0 software was used for statistical analysis. Different values of HBP between the time points for the *t*-test were generated for each group, and the time-effect relationships of BF changes were analyzed.

The level of ET-1, NO, NE in the liver tissue in each group was expressed as mean ± standard deviation. SPSS 13.0 statistical software was used for paired *t*-test of the same index between groups. *P* < 0.05 was regarded as significantly different.

## 3. Results 

### 3.1. Analyses of LSP Images of the Liver in ST36 Group

**Figure 1:**
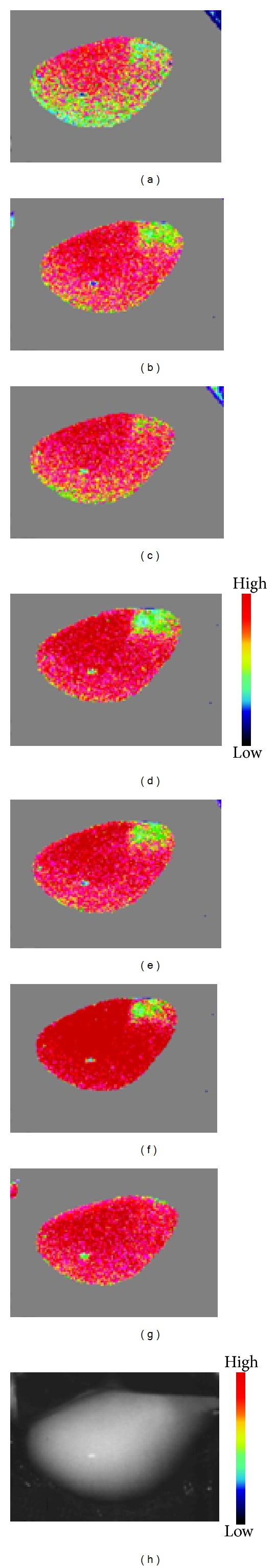
Hepatic LSP images before and after EA at ST36. (a) Before acupuncture; (b) EA for 5 min; (c) EA for 10 min; (d) EA for 15 min; (e) EA for 20 min; (f) EA for 25 min; (g) EA for 30 min; (h) digital coded brightness image of mouse liver. Before EA, LSP on the hepatic surface was displayed as light green, yellow, and red on the image. After EA for 5 min, the red areas were increased on the image immediately. When EA time was prolonged, the red areas were further extended while the light green and yellow regions gradually decreased.

In ST36 group, before EA stimulation (Figure 1(a)), more light green and yellow areas were displayed on LSP images of the liver, indicating that HBP was lower. After puncturing 5 min, red areas increased. The red area gradually added along with EA, it increased more rapidly around the hepatic portal area and much more slowly in the area close to the hepatic edge area. After 25 min (Figure 1(f)), the crimson area was found in the image; the BP reached the maximum. Afterwards, the red was lighter with an even distribution in the liver (Figure 1).

### 3.2. Analyses of LSP Images of the Liver in NA Group

**Figure 2:**
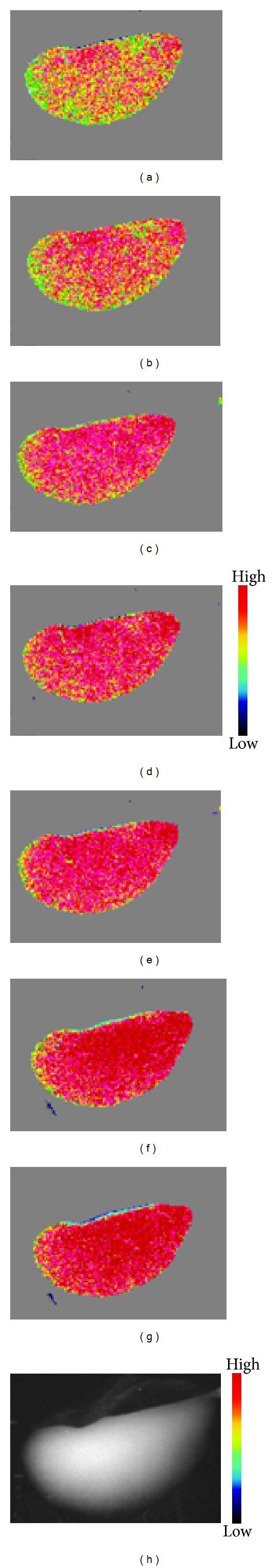
Hepatic LSP images before and after EA at nonacupoint. (a) Before acupuncture; (b) EA for 5 min; (c) EA for 10 min; (d) EA for 15 min; (e) EA for 20 min; (f) EA for 25 min; (g) EA for 30 min; (h) digital coded brightness image of mouse liver. Before EA, LSP on the hepatic surface was displayed as light green, yellow, and red on the image. After EA for 5 min, the red areas were increased on the image immediately. When EA time was prolonged, the red areas were further increased while the light green and yellow areas gradually reduced and almost disappeared.

Before and after EA, in NA group, the changes of LSP images were similar to that of ST36 group, but the range and degree of the red increase after EA were smaller than that of ST36 group (Figure 2).

### 3.3. Analyses of LSP Images of the Liver in Control Group

**Figure 3:**
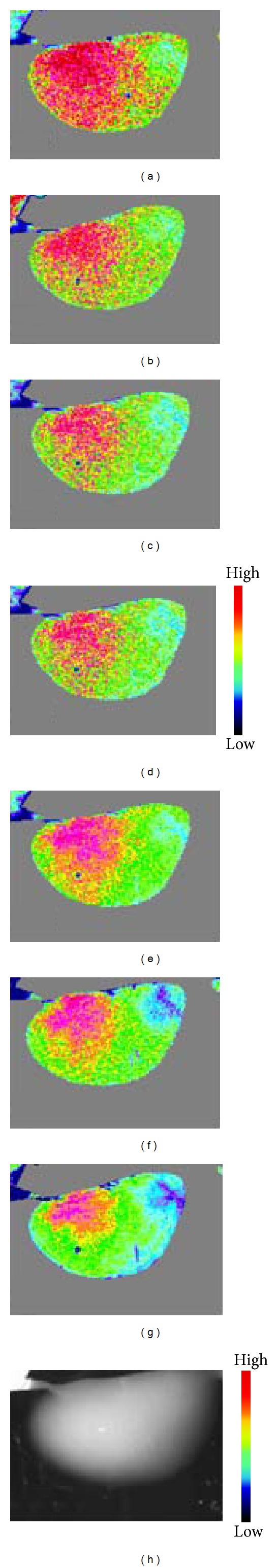
Hepatic LSP images in control group without EA. (a) At 0 min; (b) at 5 min; (c) at 10 min; (d) at 15 min; (e) at 20 min; (f) at 25 min; (g) at 30 min; (h) digital coded brightness image of mouse liver. At beginning of observation, LSP image was displayed as light green, yellow, and red on the image. Moreover the red areas gradually reduced and the yellow and greenish distribution gradually increased along with the observation time extended.

From the LSP image at the starting of investigation (Figure 3(a)), it could be seen that the HBP was rich. The HBP was high in the fan-shaped area close to hepatic portal was displayed dark red on the images, and the HBP was decreased from fan-shaped area to the liver anterior edge region were displayed yellow and light green. As investigation time passed by, the light green and yellow regions were extended, and the red areas were decreased gradually, while HBP reduced slowly (Figure 3).

### 3.4. Quantitative Analyses of HBP Changes

**Figure 4:**
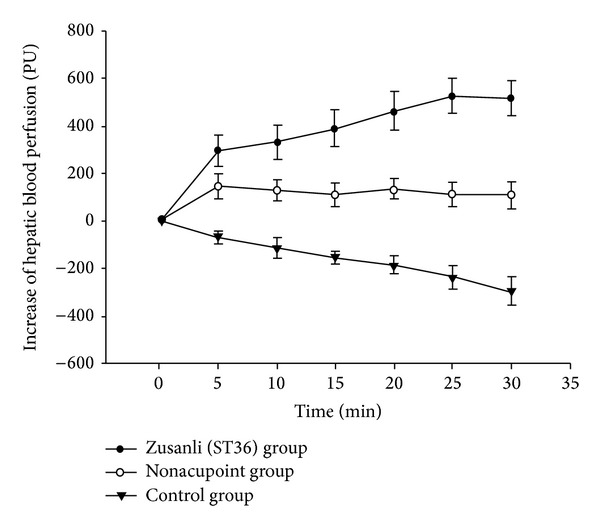
Change of the mean differences of HBP in the three groups. In ST36 or nonpoint group, 0 min was the time before EA; 5–30 min was EA. In control group, 0–30 min was monitoring continually without EA. Data are presented as mean ± SD, *n* = 20 animals for each group.

**Figure 5:**
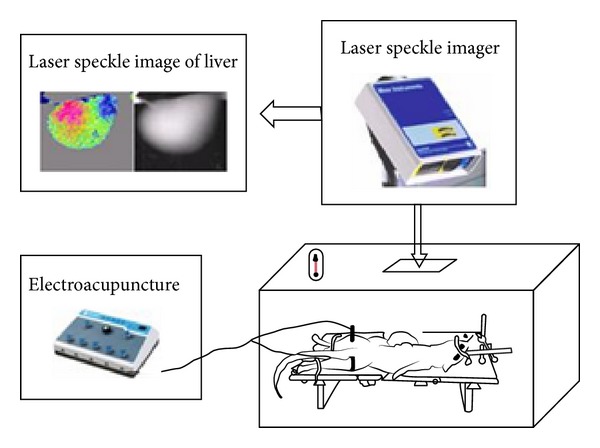
The testing method of HBP using laser speckle imaging.

HBP in each EA group was significantly increased at every time point as compared with 0 min. After 5 min, the increase of HBP was 295.54 ± 62.77 (PU), increased by 7.65 ± 1.89%. The highest increase was found at 25 min, which was 527.52 ± 75.96 PU, increased by 14.87 ± 2.35% in ST36 group. After 5 min, the increase of HBP was 88.07 ± 33.36 PU, increased by 2.26 ± 0.92%. The highest increase was found at 25 min, which was 154.04 ± 42.33 PU, increased by 4.14 ± 1.1% in NA group. In control group, HBP was decreased slowly during 30 min (Figure 4). It could be seen from Table 1 that at all time points of EA the increasing rate of HBP in NA group and control group was significantly lower than that of ST36 group (*P* < 0.05).

### 3.5. Comparison of the Levels in NO, ET-1, and NE

Data describing the levels of NO, ET-1, and NE from three groups were summarized in Table 2. The level of NO was the highest in ST36 group and the lowest in control group. Statistical analysis showed that the level of NO in ST36 group was significantly higher than control group; NA group was significantly higher than control group (*P* < 0.05). The trend was consistent with the HBP.

The level of ET-1 was the highest in control group and the lowest in ST36 group. Statistical analysis showed that the level of ET-1 in NA group and control group was significantly higher than that of ST36 group (both *P* < 0.05)

The level of NE was the highest in control group and the lowest in ST36 group; the trend was similar to the level of ET-1 change but opposite to level of NO change in each group. There were no significant differences in the level of NE among three groups.

## 4. Discussion

Liver has dual blood supply and is a huge blood bank. In it, many branches of the hepatic artery and portal veins carry the mixed blood to the hepatic sinusoids [15]. When liver functions are damaged, degeneration and necrosis of hepatocytes can cause abnormality of hepatic hemorheology and microcirculation, even in the whole body. On the other hand, abnormality of hepatic hemorheology and microcirculation can induce hepatocyte injury [16, 17]. Traditional Chinese Medicine (TCM) theory holds that “the liver stores the blood,” and the liver functions are closely related to the BF. Therefore, it is of a great significance for treatment of liver diseases to improve hepatic microcirculation. Some researches show that EA stimulation can effectively enhance the blood perfusion (BP) of pathologic tissues and organs and improve the physiological function [18–20]. It is reported that the HBP increased after acupuncture treatment detected by the Color Doppler Ultrasonography (CDU) imaging [21]. But the CDU has been applied to detect vascular distribution and BF changes of large vessels and mainly evaluate the vasculopathy and BF state of tissues or organs [22, 23]. Compared with CDU, Moor-FLPI has the following advantages: a use in investigation of microcirculation, a wide range of detection, and noncontact detection [24]. Therefore, it is very suitable to monitor BP of tissues and organs with adequate blood circulation [25, 26]. In this study, Moor-FLPI was used to display HBP in order to accurately record dynamic HBP changes after EA. The LSP images showed that HBP was stable at normal state. After EA stimulation for 30 min, the HBP continuously increased, and the effect on ST36 group was more significant than NA group. Otherwise, the vessels of hepatic portal were dense, BP increased much more in the area, and lower in the region away from the hepatic portal during EA stimulation [27].

NO is a vasodilator with stronger activity in the living body. It mediates relaxation of vascular smooth muscles and vasodilation as a second messenger. Meanwhile NO is involved in maintenance of the physiological functions of the circulatory system such as platelet adhesion and inhibition, matching between BF and ventilation of alveoli, microcirculation of glomerulus and bone marrow, regulation of cardiocerebrovascular tension, protection of myocardial cells, and so on [28–32]. ET-1 is a polypeptide with the strongest vasoconstriction activity. And it is synthesized and released by vascular endothelial cells, myocardium, smooth muscle, and so forth. It can induce vasoconstriction and decrease the blood flow by promoting Ca^2+^ influx in vascular smooth muscle cells and change the form and function of vessel walls by stimulating multiplication of vascular smooth muscle cells, extracellular matrix accumulation, and collagen synthesis [33–35]. Additionally, the functions of NO vasodilation and ET-1 vasoconstriction are antagonistic. The increase of NO would inhibit the synthesis and release of ET-1 in liver tissue [36]. NE is an important sympathetic vasoconstrictive neurotransmitter [37]. EA stimulation can activate the nervous system to adjust the synthesis and release of transmitters, just as NE dose [38, 39].

It could be seen from the results that not only was the HBP significantly increased, but also the vasoactive substances ET-1, NO, and neurotransmitter NE in liver tissue were changed after EA. Then, how did the changes of vasoactive substances in the liver relate to the increase of HBP after EA? According to the results, we think that the more the HBP after EA, the faster the hepatic BFV. Hepatic circulation was promoted by EA. As a result, the friction force between BF and vessels wall was increased. Then, the activities of vascular endothelium cells, vascular smooth muscle, and nerves were motivated by the increasing friction. Therefore, the synthesis and release of vasoactive substances and neurotransmitters must adapt to those changes. At last, in hepatic tissues, vasoconstrictive substance ET-1 and neurotransmitter NE decreased, vasodilative substance NO increased, blood vessels dilated, and the HBP improved.

ST36 was used primarily for the treatment of digestive system diseases. Clinically, ST36 was an important point in the treatment of liver disease [40–42]. In this study, at the same time point, the increasing rate of HBP and the level of NO in ST36 group were significantly higher than that of NA group and control group. And the level of the ET-1 in ST36 group was significantly lower than that of other groups (*P* < 0.05). It was suggested that the effects of EA on HC in ST36 were superior to nonacupoint. Moreover, the effect of EA on increasing HBP mainly was produced by increasing NO content and inhibiting ET-1 in liver tissue. Additionally, the mechanisms of HBP would be studied from part of vasoactive substances in this study. What would happen in the other side after EA also waits for further study.

Many clinical practices proved that acupuncture had biphasic modulation effects. It can induce abnormal physiological functions to normal levels. There are obvious differences between the effects of acupuncture on pathological state and health state. In this paper, increasing of HBP after EA was detected in health mice. It suggested that EA had an effect on HBP. Based on the result, we will study the effect of EA on HBP of liver injury model mice in the next step.

## 5. Conclusion

The effect of EA on HC was displayed using LSPI for the first time in this study. The results showed that EA at ST36 could modulate vascular activity of liver tissue and hence obviously increase HBP, providing the new animal experimental evidence for clinical effect of acupuncture on HC and mechanisms of vasoregulation. Meanwhile, it confirmed the TCM theory that acupuncture could promote Qi and blood circulation.

## Figures and Tables

**Table 1:** Comparison of the increasing rate of HBP in three groups (mean ± SD, %).

Group	*n*	0′	5 min	10 min	15 min	20 min	25 min	30 min
ST36 group	20	0	7.65 ± 1.89	8.53 ± 2.08	10.67 ± 2.25	12.68 ± 2.49	14.87 ± 2.35	14.54 ± 2.19
NA group	20	0	2.27 ± 0.92^†^	2.16 ± 0.91^†^	2.96 ± 1.10^†^	3.23 ± 1.10^†^	4.14 ± 1.10^†^	4.00 ± 1.20^†^
Control group	20	0	−1.87 ± 0.60^†^	−3.15 ± 1.10^†^	−4.25 ± 0.70^†^	−5.10 ± 1.00^†^	−6.7 ± 1.50^†^	−8.16 ± 1.60^†^

The increasing rate of HBP in the each group was counted. ^†^ *P* < 0.05, compared with ST36 group at the same time point.

**Table 2:** Comparison of levels of NO, ET-1, and NE in three groups (mean ± SD).

Group	*n*	NO (*μ*mol/gprot)	ET-1 (pg/mL)	NE (ng/mL)
ST36 group	10	70.76 ± 21.33	54.41 ± 7.13	5.30 ± 1.30
NA group	10	39.09 ± 17.83*	121.09 ± 31.07^†^	8.36 ± 1.06
Control group	10	37.01 ± 14.23*	137.81 ± 24.30^†^	19.27 ± 7.57

The data of NO, ET-1, and NE contents in liver tissue in the three groups were in Table 2. *P < 0.05, compared with ST36 group; ^†^ *P* < 0.05, compared with ST36 group.
